# Associations Between Rural or Urban Status, Health Outcomes and Behaviors, and COVID-19 Perceptions Among Meditation App Users: Longitudinal Survey Study

**DOI:** 10.2196/26037

**Published:** 2021-05-26

**Authors:** Nishat Bhuiyan, Megan Puzia, Chad Stecher, Jennifer Huberty

**Affiliations:** 1 College of Health Solutions Arizona State University Phoenix, AZ United States; 2 Behavioral Research and Analytics, LLC Salt Lake City, UT United States

**Keywords:** mHealth, rural health, physical activity, mental health, COVID-19

## Abstract

**Background:**

Rural and urban differences in health outcomes and behaviors have been well-documented, with significant rural health disparities frequently highlighted. Mobile health (mHealth) apps, such as meditation apps, are a novel method for improving health and behaviors. These apps may be a critical health promotion strategy during the COVID-19 pandemic and could potentially be used to address rural health disparities. However, limited research has assessed whether meditation app health outcomes are associated with rural and urban residence, and it is unclear whether disparities in health and behaviors between rural and urban populations would persist among meditation app users.

**Objective:**

We aimed to explore associations between rural or urban status, psychological outcomes, and physical activity among users of a mobile meditation app. We further aimed to explore associations between rural or urban status and perceived effects of COVID-19 on stress, mental health, and physical activity, and to explore changes in these outcomes in rural versus urban app users over time.

**Methods:**

This study was a secondary analysis of a national survey conducted among subscribers to the meditation app Calm. Eligible participants completed online baseline surveys from April to June 2020, and follow-up surveys from June to September 2020, assessing demographics, psychological outcomes, physical activity, and perceived effects of COVID-19 on stress, mental health, and physical activity.

**Results:**

Participants (N=8392) were mostly female (7041/8392, 83.9%), non-Hispanic (7855/8392, 93.6%), and White (7704/8392, 91.8%); had high socioeconomic status (income ≥US $100,000: 4389/8392, 52.3%; bachelor’s degree or higher: 7251/8392, 86.4%); and resided in a metropolitan area core (rural-urban commuting area code 1: 7192/8392, 85.7%). Rural or urban status was not associated with baseline stress, depression, anxiety, pre–COVID-19 and current physical activity, or perceived effects of COVID-19 on stress, mental health, and physical activity. Repeated-measures models showed overall decreases in depression, anxiety, and perceived effects of COVID-19 on physical activity from baseline to follow-up, and no significant changes in stress or perceived effects of COVID-19 on stress and mental health over time. Models also showed no significant main effects of rural or urban status, COVID-19 statewide prevalence at baseline, or change in COVID-19 statewide prevalence.

**Conclusions:**

We did not find associations between rural or urban status and psychological outcomes (ie, stress, depression, and anxiety), physical activity, or perceived effects of COVID-19 on stress, mental health, and physical activity. Rural or urban status does not appear to drive differences in outcomes among meditation app users, and the use of mHealth apps should continue to be explored as a health promotion strategy in both rural and urban populations. Furthermore, our results did not show negative cumulative effects of COVID-19 on psychological outcomes and physical activity among app users in our sample, the majority of whom were urban, White, female, and of high socioeconomic status. Further research is needed to investigate meditation app use as a health promotion strategy in rural and urban populations.

## Introduction

Significant rural and urban status–related differences in health outcomes and behaviors have been well-documented in the United States [1-6]. Some studies have demonstrated poorer outcomes in urban areas, such as increased risk for certain mental disorders and cancer incidence [1,2]. However, for the majority of health outcomes and behaviors, rural residents face poorer outcomes compared to their urban counterparts, including higher chronic disease incidence and mortality rates and lower diet quality and physical activity [3-6], underscoring the need for methods to address rural health disparities.

The use of mobile health (mHealth) apps has been rapidly growing as a novel method for health promotion [7,8], as strategies that include mHealth apps may be more cost-effective and scalable as well as have wider reach than in-person–delivered programs [9]. mHealth tools may be even more critical during the current COVID-19 pandemic, given the limited availability and potential risks associated with in-person–delivered programs [10]. Studies have demonstrated that mHealth apps can effectively improve mental and physical health and behaviors [7,8]. Rural areas in the United States are often characterized by limitations in resources, such as medical facilities and specialty health care services; thus, the use of mHealth apps to remotely deliver health interventions to improve outcomes in rural populations may be of paramount importance to rural communities and help to address ongoing rural health disparities [11,12].

The use of meditation apps, in particular, could also be used to potentially address disparities in mental health outcomes and health behaviors faced by rural residents. Meditation is a well-known strategy for improving mental health outcomes, such as stress, depression, and anxiety [13,14]. Furthermore, growing literature further suggests that meditation is a promising tool for counteracting sedentariness to address physical inactivity [15]. Given the poorer mental health outcomes [16,17], lower access to mental health treatment [17], and lower prevalence of positive health outcomes and behaviors among rural residents [18], research exploring rural and urban status–related differences in health and behavior outcomes among meditation app users is warranted.

Limited research has assessed whether mHealth app outcomes are associated with rural and urban residence, and it is currently unclear whether disparities in health and behaviors between rural and urban populations would persist among mobile meditation app users. Thus, the purpose of this study is to (1) explore associations between rural or urban status, psychological outcomes, and physical activity; (2) explore associations between rural or urban status and perceived effects of COVID-19 on stress, mental health, and physical activity; and (3) assess changes in psychological outcomes, physical activity, and perceived effects of COVID-19 over time among rural versus urban users of a mobile meditation app.

## Methods

### Overview

This study was a secondary analysis of a national survey conducted in a nonrandom convenience sample of paying subscribers to the mobile meditation app Calm. All study materials and procedures were reviewed and approved by the Institutional Review Board at Arizona State University (protocol ID: STUDY00014534). Potentially eligible subscribers met the following inclusion criteria: (1) were 18 years of age or older, (2) had opened an email from Calm and used Calm at least once in the last 90 days, (3) were able to read and understand English, and (4) were United States residents. Potentially eligible subscribers were identified by the Calm informatics team and were sent a study recruitment email by the research team. The recruitment email included a brief study description and the link to an online Qualtrics eligibility survey to verify that they met the following inclusion criteria: (1) were at least 18 years of age, (2) were able to read and understand English, and (3) resided in the United States or a United States territory. This survey was free and voluntary and took approximately 3 minutes to complete.

Eligible participants were emailed a link to complete an electronic informed consent form, which stated the study purpose; the identity of the investigator; the length of time to complete the survey; which data were stored, where, and for how long; potential risks and benefits; and compensation details. Eligible participants were emailed links to online Qualtrics baseline and follow-up surveys. Surveys took approximately 15 minutes to complete and were free and voluntary; in addition, participants were able to skip survey questions. 

The time frame for this study was approximately 2 months, from baseline to follow-up. Baseline surveys were distributed from April 22 to June 3, 2020, and follow-up surveys were distributed from June 26 to September 11, 2020. To maintain participant confidentiality, study data were imported into a secure and backed-up MySQL (Structured Query Language) database. Each database user had their own username and password, with permissions set appropriately for that user. Query logging was enabled to provide an electronic audit trail that recorded all user interactions with the database. The database did not store personally identifiable information; instead, records were linked to individual participants via a unique participant ID. Regarding compensation, all participants were entered into a random drawing for 1 of 20 gift cards valued at US $50.00 for completing the baseline survey (ie, 20 participants received US $50.00 at baseline) and a random drawing for 1 of 20 gift cards valued at US $50.00 for completing the follow-up survey (ie, 20 participants received US $50.00 at follow-up).

### Measures

The online Qualtrics baseline and follow-up surveys included both investigator-developed and validated questionnaires. Participants self-reported demographic characteristics; stress, via the Perceived Stress Scale [19]; depression and anxiety, via the Hospital Anxiety and Depression Scale (HADS) [20]; pre–COVID-19 and current physical activity (days/week); and the extent to which COVID-19 affected their stress, mental health, and physical activity. The Perceived Stress Scale includes 10 items that measure the degree of self-appraised stress in one’s life within the past month [19]. Response items are scored on a 5-point Likert scale ranging from 0 (never) to 4 (very often). Items are summed to produce a total score ranging from 0 to 40, with higher scores indicating higher levels of perceived stress. The Perceived Stress Scale is a reliable and valid measure that has demonstrated good internal consistency (Cronbach α=.74-.91) [19]. The HADS is a 14-item scale measuring levels of anxiety and depression. Seven items comprise the anxiety subscale (HADS-A) and seven items comprise the depression subscale (HADS-D) [20]. Response items are scored on a 4-point Likert scale ranging from 0 to 3. Items are summed to produce a total score ranging from 0 to 21 for each subscale. Scores between 0 and 7 are considered normal, scores from 8 to 10 are considered borderline abnormal, and scores from 11 to 21 are considered abnormal. The HADS is a valid and reliable tool, with internal consistency reported to reach α levels of .93 and .90 for the HADS-A and HADS-D subscales, respectively [20]. Participants were asked to self-report how many days per week (on a scale of 0 to 7) of physical activity they participated in prior to COVID-19 as well as their current participation. Participants were asked, via investigator-developed items, “To what extent do you feel the COVID-19 pandemic has affected your stress?” “To what extent do you feel the COVID-19 pandemic has affected your mental health?” and “To what extent do you feel the COVID-19 pandemic has affected your physical activity?” Response items were scored on a 5-point Likert scale, ranging from 0 (not at all) to 4 (very much), with higher summed scores representing a greater impact of COVID-19 on outcomes.

Participant ZIP Codes were categorized into levels of urbanization using the US Department of Agriculture’s rural-urban commuting area (RUCA) codes, which range from 1, indicating a metropolitan core area, to 10, indicating a rural area [21]. RUCA code definitions can be found in Table 1. To define rural or urban status, a binary variable was created by dummy-coding RUCA 1 as urban and RUCA 2 to 10 as rural. As an alternative approach, we also treated the RUCA code as a continuous variable in replicated study analyses, the results of which can be found in Multimedia Appendix 1.

COVID-19 prevalence data were derived from the US Centers for Disease Control and Prevention aggregate data set of daily numbers of confirmed and probable case and deaths over time [22]. This information was used to categorize each state’s relative COVID-19 risk, where low-prevalence COVID-19 states were defined as those with fewer than 10,000 cases per 100,000 at the time of survey distribution and high-prevalence COVID-19 states were those with 10,000 cases or more per 100,000. At baseline, high-prevalence COVID-19 states included California, Colorado, Connecticut, Florida, Georgia, Illinois, Indiana, Louisiana, Massachusetts, Maryland, Michigan, New Jersey, New York, Ohio, Pennsylvania, Texas, Virginia, and Washington. At follow-up, high-prevalence COVID-19 states additionally included Alabama, Arkansas, Arizona, DC, Delaware, Iowa, Kansas, Kentucky, Minnesota, Missouri, Mississippi, North Carolina, Nebraska, New Mexico, Nevada, Oklahoma, Rhode Island, South Carolina, Tennessee, Utah, and Wisconsin. A binary variable, *COVID-19 state baseline*, was created based on high-prevalence COVID-19 states, and a second variable, *COVID-19 state change*, was created based on states that were low prevalence at baseline and changed to high prevalence at follow-up.

**Table 1:** Participant demographic characteristics.

Characteristic		Value (N=8392)
Age (years), mean (SD)		47.53 (13.83)
**Gender (n=7303), n (%)**		
	Female	6129 (83.92)
	Male	1147 (15.71)
	Other	27 (0.37)
**Ethnicity (n=6774), n (%)**		
	Hispanic	436 (6.44)
	Non-Hispanic	6338 (93.56)
**Race (n=7338), n (%)**		
	White	6586 (91.75)
	Black or African American	231 (3.22)
	Asian	216 (3.01)
	Native American or Alaska Native	83 (1.16)
	Native Hawaiian or Pacific Islander	27 (0.38)
	Other	195 (2.72)
**Income (US $) (n=6949), n (%)**		
	≤20,000	212 (3.05)
	21,000-40,000	402 (5.79)
	41,000-60,000	705 (10.15)
	61,000-80,000	942 (13.56)
	81,000-100,000	1055 (15.18)
	>100,000	3633 (52.28)
**Education level (n=7319), n (%)**		
	11^th^ grade or less	8 (0.11)
	High school or General Educational Development	161 (2.20)
	Some college	826 (11.29)
	2-year college or technical degree	2670 (36.48)
	Bachelor’s degree	424 (5.79)
	Graduate degree	3230 (44.13)
**Employment (n=7297), n (%)**		
	Employed	5084 (69.67)
	Retired	1012 (13.87)
	Unemployed	477 (6.54)
	Homemaker	306 (4.19)
	Unable to work	252 (3.45)
	Student	166 (2.27)
**Rural-urban commuting area (RUCA) code (n=7044)** **, n (%)**		
	1: *Metropolitan area core: primary flow within an Urbanized Area (UA)*	6038 (85.73)
	2: *Metropolitan area high commuting: primary flow 30% or more to a UA*	409 (5.83)
	3: *Metropolitan area low commuting: primary flow 10% to 30% to a UA*	23 (0.29)
	4: *Micropolitan area core: primary flow within an Urban Cluster (UC) of 10,000 to 49,999 (large UC)*	295 (4.18)
	5: *Micropolitan high commuting: primary flow 30% or more to a large UC*	42 (0.63)
	6: *Micropolitan low commuting: primary flow 10% to 30% to a large UC*	9 (0.11)
	7: *Small town core: primary flow within a UC of 2500 to 9999 (small UC*)	121 (1.69)
	8: *Small town high commuting: primary flow 30% or more to a small UC*	11 (0.24)
	9: *Small town low commuting: primary flow 10% to 30% to a small UC*	11 (0.22)
	10: *Rural areas: primary flow to a tract outside a UA or UC*	85 (1.19)

### Statistical Analysis

Descriptive statistics and frequencies were computed to describe sample characteristics. Unadjusted and adjusted regression models were used to examine the association between rural or urban status (ie, RUCA code) and (1) psychological outcomes (ie, stress, depression, and anxiety), (2) pre–COVID-19 and current physical activity, and (3) perceived effects of COVID-19 on stress, mental health, and physical activity, controlling for demographics (ie, gender, age, ethnicity, race, income, education, employment, self-reported Calm app use, and statewide COVID-19 prevalence at baseline). To assess changes in outcomes over time, repeated-measures analyses of variance (ANOVAs) were used. Models included baseline and follow-up outcomes (ie, stress, depression, anxiety, effect of COVID-19 on stress, effect of COVID-19 on mental health, and effect of COVID-19 on physical activity) as within-subjects factors, rural and urban statuses as between-subjects factors, and gender, age, ethnicity, race, income, education, employment, self-reported Calm app use, COVID-19 state baseline (ie, high statewide COVID-19 prevalence at baseline), and COVID-19 state change (ie, change from low statewide COVID-19 prevalence at baseline to high statewide COVID-19 prevalence at follow-up) as covariates. To further assess the effect of rural or urban status and changes in statewide COVID-19 prevalence on changes in outcomes over time, we tested a three-way interaction with time, COVID-19 statewide prevalence, and rural or urban status. All statistical analyses were performed using SPSS, version 26.0 (IBM Corp), with significance inferred at *P*<.05.

## Results

There were total of 8392 participants. Most participants were female (7041/8392, 83.9%), non-Hispanic (7855/8392, 93.6%), and White (7704/8392, 91.8%); had high socioeconomic status (income ≥US $100,000: 4389/8392, 52.3%; bachelor’s degree or higher: 7251/8392, 86.4%); and resided in a metropolitan area core (RUCA 1: 7192/8392, 85.7%) (Table 1).

As shown in Table 2, at baseline, participants reported moderate stress (mean score 18.12, SD 6.29), where a score of 14 to 26 is considered moderate [23,24]; borderline abnormal levels of depression (mean score 8.89, SD 4.12), where a score of 8 to10 is considered borderline abnormal [20]; normal levels of anxiety (mean score 5.87, SD 3.61), where a score of 0 to 7 is considered normal [20]; and being physically active (mean 4.89 days/week, SD 2.29).

**Table 2:** Baseline psychological outcomes, physical activity, and perceived effects of COVID-19.

Outcome, physical activity, or effect		Mean (SD)
**Psychological outcome**		
	Stress score^a^	18.12 (6.29)
	Depression score^b^	8.89 (4.12)
	Anxiety score^b^	5.87 (3.61)
**Physical activity (days/week)^c^**		
	Pre–COVID-19 physical activity	4.87 (2.04)
	Current physical activity	4.89 (2.29)
**Perceived effect^d^ of COVID-19 on:**		
	Stress	1.82 (0.80)
	Mental health	2.07 (0.83)
	Physical activity	2.72 (1.29)

^a^Stress was measured using the Perceived Stress Scale; summed scores range from 0 to 40, with higher scores indicating higher levels of perceived stress.

^b^Depression and anxiety were measured using the depression and anxiety subscales, respectively, of the Hospital Anxiety and Depression Scale; summed scores range from 0 to 21 (normal: 0-7; borderline abnormal: 8-10; abnormal: 11-21).

^c^Participants self-reported how many days per week (on a scale of 0 to 7) of physical activity they participated in.

^d^Participants responded to investigator-developed items; response scores range from 1 to 5, with higher scores indicating higher perceived effects of COVID-19.

As shown in Table 3, in both unadjusted and adjusted regression models, rural or urban status was not significantly associated with baseline stress, depression, anxiety, physical activity, or perceived effects of COVID-19 on stress, mental health, and physical activity. In our alternative analyses where we treated RUCA as a continuous variable, we also found no significant associations between rural or urban status and mental health outcomes, physical activity, or perceived effects of COVID-19 (Multimedia Appendix 1).

**Table 3:** Unadjusted and adjusted regression models exploring associations between rural or urban status and psychological outcomes, physical activity, and perceived effects of COVID-19.

Model				Rural or urban difference, β (SE)	*P* value
**Unadjusted model**					
	**Psychological outcome**				
		Stress	0.30 (0.22)		.09
		Depression	0.15 (0.14)		.29
		Anxiety	–0.15 (0.12)		.24
	**Physical activity**				
		Pre–COVID-19 physical activity	0.08 (0.07)		.27
		Current physical activity	0.08 (0.08)		.31
	**Perceived effect of COVID-19 on:**				
		Stress	–0.05 (0.03)		.10
		Mental health	–0.07 (0.03)		.11
		Physical activity	–0.05 (0.04)		.29
**Adjusted model^a^**					
	**Psychological outcome**				
		Stress	0.02 (0.22)		.91
		Depression	0.07 (0.15)		.64
		Anxiety	–0.24 (0.13)		.07
	**Physical activity**				
		Pre–COVID-19 physical activity	0.12 (0.08)		.10
		Current physical activity	0.16 (0.09)		.05
	**Perceived effect of COVID-19 on:**				
		Stress	–0.05 (0.03)		.09
		Mental health	–0.07 (0.03)		.22
		Physical activity	–0.02 (0.05)		.67

^a^The model controlled for gender, age, ethnicity, race, income, education, employment, Calm app use, and statewide COVID-19 prevalence at baseline.

As shown in Table 4, results from repeated-measures ANOVAs showed no significant changes in stress or perceived effects of COVID-19 on stress and mental health from baseline to follow-up. In models of depression, anxiety, and effect of COVID-19 on physical activity, there were significant main effects of time, which showed that, overall, symptoms of depression, anxiety, and perceived effect of COVID-19 on physical activity decreased from baseline to follow-up. Furthermore, there were significant main effects of age on most outcomes, which showed that, at baseline, older participants had greater stress, depression, and anxiety; engaged in more physical activity; and perceived that COVID-19 had a greater effect on their stress and mental health. There were also significant time × age interactions in models of stress and anxiety, such that older participants had smaller decreases in stress and anxiety over time than did younger participants. Results of full models with covariates can be found in Multimedia Appendix 1.

There were no significant main effects of rural or urban status, statewide COVID-19 prevalence at baseline, or change in statewide COVID-19 prevalence in any of the models (Table 4). In addition, there were no significant time × rural or urban status interactions, time × statewide COVID-19 prevalence at baseline interactions, or time × change in statewide COVID-19 prevalence interactions (Table 4). Lastly, we found no significant three-way interactions (ie, time × statewide COVID-19 prevalence at baseline × rural or urban status and time × change in statewide COVID-19 prevalence × rural or urban status).

**Table 4:** Results of repeated-measures analyses of variance for baseline and follow-up study outcomes^a^.

Model			Baseline score, mean (SD)		Follow-up score, mean (SD)		*F* test (*df*)		*P* value
**Stress**			18.013 (6.174)		17.180 (6.322)				
	Time					0.049 (1, 2636)		.83	
	Rural-urban commuting area (RUCA)					0.160 (1, 2636)		.69	
	Time × RUCA					0.000 (1, 2636)		.99	
	COVID-19 state baseline					1.078 (1, 2636)		.30	
	Time × COVID-19 state baseline					0.015 (1, 2636)		.90	
	Time × COVID-19 state baseline × RUCA					0.116 (1, 2636)		.73	
	COVID-19 state change					0.069 (1, 2636)		.79	
	Time × COVID-19 state change					0.006 (1, 2636)		.94	
	Time × COVID-19 state change × RUCA					0.168 (1, 2636)		.68	
**Depression**			8.832 (4.097)		8.396 (4.156)				
	Time					3.868 (1, 2613)		.049	
	RUCA					0.017 (1, 2613)		.90	
	Time × RUCA					0.339 (1, 2613)		.56	
	COVID-19 state baseline					0.386 (1, 2613)		.54	
	Time × COVID-19 state baseline					0.112 (1, 2613)		.74	
	Time × COVID-19 state baseline × RUCA					0.047 (1, 2613)		.83	
	COVID-19 state change					0.038 (1, 2613)		.85	
	Time × COVID-19 state change					0.519 (1, 2613)		.47	
	Time × COVID-19 state change × RUCA					0.183 (1, 2613)		.67	
**Anxiety**			5.755 (3.590)		5.474 (3.663)				
	Time					4.648 (1, 2613)		.03	
	RUCA					0.409 (1, 2613)		.52	
	Time × RUCA					0.353 (1, 2613)		.55	
	COVID-19 state baseline					0.022 (1, 2613)		.88	
	Time × COVID-19 state baseline					0.626 (1, 2613)		.43	
	Time × COVID-19 state baseline × RUCA					1.121 (1, 2613)		.29	
	COVID-19 state change					0.211 (1, 2613)		.65	
	Time × COVID-19 state change					0.010 (1, 2613)		.92	
	Time × COVID-19 state change × RUCA					0.310 (1, 2613)		.58	
**Physical activity**			4.930 (2.305)		4.950 (2.243)				
	Time					0.188 (1, 2519)		.67	
	RUCA					1.227 (1, 2519)		.27	
	Time × RUCA					1.248 (1, 2519)		.26	
	COVID-19 state baseline					0.046 (1, 2519)		.83	
	Time × COVID-19 state baseline					0.659 (1, 2519)		.42	
	Time × COVID-19 state baseline × RUCA					0.779 (1, 2519)		.38	
	COVID-19 state change					0.000 (1, 2519)		>.99	
	Time × COVID-19 state change					2.061 (1, 2519)		.15	
	Time × COVID-19 state change × RUCA					3.550 (1, 2519)		.06	
**Effect of COVID-19 on stress**			1.830 (0.821)		1.790 (0.783)				
	Time					0.717 (1, 2487)		.40	
	RUCA					0.189 (1, 2487)		.66	
	Time × RUCA					3.344 (1, 2487)		.07	
	COVID-19 state baseline					0.935 (1, 2487)		.33	
	Time × COVID-19 state baseline					2.506 (1, 2487)		.11	
	Time × COVID-19 state baseline × RUCA					1.077 (1, 2487)		.30	
	COVID-19 state change					0.002 (1, 2487)		.97	
	Time × COVID-19 state change					1.203 (1, 2487)		.27	
	Time × COVID-19 state change × RUCA					0.084 (1, 2487)		.77	
**Effect of COVID-19 on mental health**			2.110 (0.846)		2.030 (0.821)				
	Time					0.081 (1, 2481)		.78	
	RUCA					0.138 (1, 2481)		.71	
	Time × RUCA					0.002 (1, 2481)		.96	
	COVID-19 state baseline					0.123 (1, 2481)		.73	
	Time × COVID-19 state baseline					0.255 (1, 2481)		.61	
	Time × COVID-19 state baseline × RUCA					0.510 (1, 2481)		.48	
	COVID-19 state change					0.069 (1, 2481)		.79	
	Time × COVID-19 state change					0.006 (1, 2481)		.94	
	Time × COVID-19 state change × RUCA					0.168 (1, 2481)		.68	
**Effect of COVID-19 on physical activity**			2.740 (1.278)		2.670 (1.261)				
	Time					3.868 (1, 2483)		.049	
	RUCA					0.017 (1, 2483)		.90	
	Time × RUCA					0.339 (1, 2483)		.56	
	COVID-19 state baseline					0.386 (1, 2483)		.54	
	Time × COVID-19 state baseline					0.112 (1, 2483)		.74	
	Time × COVID-19 state baseline × RUCA					0.047 (1, 2483)		.83	
	COVID-19 state change					0.038 (1, 2483)		.85	
	Time × COVID-19 state change					0.519 (1, 2483)		.47	
	Time × COVID-19 state change × RUCA					0.183 (1, 2483)		.67	

^a^Baseline and follow-up mean (SD) values were only reported for each within-subjects factor, while *F* test (*df*) values and *P* values were only reported for each between-subjects factor and interaction effects.

## Discussion

### Principal Findings

In this study, we explored associations between rural or urban status, psychological outcomes, and physical activity among meditation app users. We additionally explored associations between rural or urban status and perceived effects of the ongoing COVID-19 pandemic on stress, mental health, and physical activity, as well as changes in study outcomes among rural versus urban app users over time. Overall, we found no significant associations between rural or urban status, psychological outcomes, physical activity, or perceived effects of COVID-19 on stress, mental health, and physical activity at baseline. We also found that there were significant decreases in depression, anxiety, and perceived effects of COVID-19 on physical activity over time, but there were no significant changes in stress, physical activity participation, or perceived effects of COVID-19 on stress or mental health. Additionally, there were no significant time × statewide COVID-19 prevalence × rural or urban status interactions.

Our findings generally contrast previous studies that demonstrated rural and urban differences in physical and mental health outcomes and behaviors [1-6]. Specifically, some studies found a higher prevalence of major mental disorders, including mood and anxiety disorders, in urban areas [2,25]. For example, one literature review concluded that living in urban cities was associated with a considerably higher risk for schizophrenia [2]; in addition, another meta‐analysis of urban and rural differences in psychiatric disorders, which was conducted on data taken from 20 population survey studies, found that prevalence rates for psychiatric disorders were significantly higher in urban areas compared with rural areas [25]. Other studies found a higher prevalence of depression in rural areas [16] and have shown that rural residents receive less mental health treatment despite poorer mental health [17]. For example, a cross-sectional study using the National Health Interview Survey found that depression prevalence was significantly higher among rural populations than among urban populations [16]. Another study using data from the Medical Expenditure Panel Survey concluded that the odds of receiving any mental health treatment and specialized mental health treatment were 47% and 72% higher, respectively, for metropolitan residents compared to those living in the most rural settings [17].

Regarding health behaviors such as physical activity, rural residents have consistently been less physically active compared to their urban counterparts, which has been attributed to increased barriers and limited physical activity resources in rural areas [6,18,26]. For example, an analysis of data from the Behavioral Risk Factor Surveillance System, conducted among 398,208 adults, demonstrated that residents of nonmetropolitan counties had a lower prevalence of meeting national physical activity guidelines compared to their metropolitan counterparts [18]. These referenced studies exploring rural and urban differences in health outcomes and behaviors have generally been conducted among adults outside the context of participation in a behavioral health intervention or use of an available behavioral health program, unlike this study, which explored these differences among users of a meditation app.

This was the first study, to our knowledge, to assess rural and urban differences among meditation app users during the COVID-19 pandemic. Our results suggest that the rural residents in our sample who use meditation apps have access to tools that may address rural health disparities. For example, meditation app users need to own a mobile device and have internet access, which are social determinants of health [27]. Furthermore, mobile meditation app users likely possess digital literacy (ie, the ability to understand and utilize electronic resources or identify, access, and use electronic information from networks as well as having the skills to decipher texts, sounds, or images) [28,29]. Lastly, seeking out, downloading, and using a meditation app to manage health-related outcomes and symptoms reflect health literacy (ie, the capacity to obtain, process, and understand basic health information and services needed to make appropriate health decisions) [30], which is associated with mHealth app use [31]. Overall, a combination of mobile device ownership, internet access, and digital and health literacy may be important tools for addressing rural health disparities. The use of a meditation app, specifically, may further contribute to addressing disparities via improvement of mental health outcomes and health behaviors among rural residents. Thus, research should continue to explore and establish the use of meditation apps for health promotion in both rural and urban populations.

There were no associations between rural or urban status and perceived effects of the COVID-19 pandemic on stress, mental health, or physical activity. Emerging literature has demonstrated that stress and symptoms of anxiety and depression are common psychological responses to COVID-19 [32], but studies have not yet focused on rural populations or assessed rural and urban differences in perceived effects of COVID-19. Our results suggest that during this ongoing pandemic, mobile device ownership, internet access, digital and health literacy, and meditation app use may address disparities related to the perceived effects of COVID-19 on health and health behaviors. This further reinforces the importance of using meditation apps among both rural and urban populations. However, it is important to note that our sample largely consisted of non-Hispanic White women with relatively high socioeconomic status, and may not be reflective of rural or urban mHealth app users from lower-income or racial and ethnic minority backgrounds, and the benefits of meditation apps and their potential for reducing disparities must be confirmed with studies in these underrepresented populations. Furthermore, given that the majority of our sample was urban, the absence of rural and urban differences in outcomes must be confirmed in a larger, more representative sample.

We found overall decreases in depression, anxiety, and perceived effect of COVID-19 on physical activity and no overall changes from baseline to follow-up in stress, physical activity, or perceived effects of COVID-19 on stress and mental health. Prior studies have generally demonstrated a reduction in physical activity since the start of the pandemic [33-35], and our results suggest that meditation apps may be a potential strategy for counteracting decreases in physical activity during the pandemic. Studies assessing changes in psychological outcomes over the course of the COVID-19 pandemic have resulted in mixed findings; for example, one study found increases in depression and decreases in anxiety in Argentina [36], another study found increases in anxiety in the United Kingdom [37], and another found no changes in stress, depression, or anxiety in China [38]. However, there have been limited studies longitudinally assessing changes in mental health outcomes during the pandemic in the United States. Our results provide preliminary evidence that despite the increase in state-level COVID-19 cases in the United States, there may not be negative cumulative effects of the pandemic on psychological outcomes and health behaviors among our sample (ie, mainly urban, White, female, and of high socioeconomic status) of meditation app users. However, further studies are necessary to understand longer-term effects of COVID-19 in this group, and to establish whether meditation delivered via mobile apps is an effective strategy for promoting health outcomes and behaviors over the course of the pandemic. Our results further suggest no significant effect of rural or urban status and statewide COVID-19 prevalence—both at baseline and change from baseline to follow-up—on changes in outcomes over time. However, more research is needed to elucidate time-varying changes in psychological and behavioral outcomes between rural and urban meditation app users, which could help to identify at-risk groups for interventions.

### Limitations

This study was the first to explore associations between rural or urban status and psychological outcomes, physical activity, and perceived effects of COVID-19 on stress, mental health, and physical activity among meditation app users. However, there were limitations that should be noted. Our sample was primarily female, non-Hispanic, and White and had high socioeconomic status, limiting generalizability of findings to other populations, such as racial and ethnic minorities and low-income individuals. The majority of our sample was further categorized as urban, with 85.7% of participants residing in the US Department of Agriculture’s definition of a metropolitan core area (ie, RUCA 1). Although these sample demographics are frequently reflected in research assessing rural and urban differences, with studies including 80.0% to 88.7% of participants residing in RUCA 1 or the highest level of urbanization [3,39-41], future research in this area should aim to include a larger proportion of rural residents.

Furthermore, all outcomes in this study were assessed via self-report measures, which are subject to social desirability and recall bias. In addition, another limitation was the use of investigator-developed survey questions as opposed to validated questionnaires. However, for some outcomes, such as the extent to which COVID-19 impacted outcomes, there were no validated instruments when this study was conducted. With regard to behavioral outcomes (ie, physical activity and meditation), future studies in this area should aim to use device-based measures. Physical activity, in particular, is one of the outcomes to be particularly cautious about when interpreting study results. Physical activity is often overreported by participants in research studies, with respondents reporting higher rates of, or more frequent, activity than actual behavior warrants, which in turn causes self-reported measures of physical activity to suffer from low validity [42]. Thus, given our self-reported items for measuring physical activity in this study, it is important to note that these results are preliminary and must be validated in larger studies using device-based measures, which may be complemented with self-report measures to provide activity type and context. Another limitation was inherent to the study design, which included a national survey conducted among a nonrandom convenience sample. Although this study was able to contribute novel, preliminary findings, future research should assess associations between rural or urban status and health outcomes among mHealth app users in a randomized controlled trial in order to decrease bias and increase scientific rigor. Lastly, although we included total state-level COVID-19 cases over time in the analyses, a combination of additional factors, such as lockdowns, hospitalizations, and deaths, may have had an influence on participants’ psychological and physical activity outcomes and perceived effects of COVID-19 on health and behaviors. Further research is also needed to establish whether cumulative effects of the pandemic will become more apparent after a longer period of time.

### Conclusions

Overall, we did not find any associations between rural or urban status and psychological outcomes (ie, stress, depression, and anxiety), pre–COVID-19 and current physical activity, or perceived effects of COVID-19 on stress, mental health, and physical activity among users of a meditation app. Rural or urban status does not appear to drive differences in outcomes among meditation app users, and the use of mHealth apps should continue to be explored as a health promotion strategy in both rural and urban populations. Our results also did not show negative cumulative effects of COVID-19 on psychological outcomes and physical activity in our sample, and research should continue to explore meditation apps as a health promotion strategy during the pandemic.

## Appendix

Multimedia Appendix 1 Unadjusted and adjusted regression models exploring associations between rural-urban status (ie, continuous rural-urban commuting area [RUCA]) and mental health outcomes, physical activity, and perceived effects of COVID-19 as well as results of repeated measures analyses.

## Abbreviations

ANOVA: analysis of variance
HADS: Hospital Anxiety and Depression Scale
HADS-A: Hospital Anxiety and Depression Scale–anxiety subscale
HADS-D: Hospital Anxiety and Depression Scale–depression subscale
mHealth: mobile health
RUCA: rural-urban commuting area
SQL: Structured Query Language
